# Community engagement during COVID: A field report from seven CTSAs

**DOI:** 10.1017/cts.2021.785

**Published:** 2021-04-27

**Authors:** Erica E. Marsh, Michael D. Kappelman, Rhonda G. Kost, Gia Mudd-Martin, Jackilen Shannon, Louisa A. Stark, Olveen Carrasquillo

**Affiliations:** 1 Michigan Institute for Clinical and Health Research, University of Michigan, Ann Arbor, MI, USA; 2 North Carolina Translational and Clinical Sciences (NCTraCS) Institute, University of North Carolina, Chapel Hill, NC, USA; 3 The Rockefeller University (RU) Center for Clinical and Translational Science (CCTS), New York, NY, USA; 4 The University of Kentucky Center for Clinical and Translational Science, Lexington, KY, USA; 5 Oregon Clinical and Translational Research Institute, Oregon Health & Science University, Portland, OR, USA; 6 Center for Clinical and Translational Science, University of Utah, Salt Lake City, UT, USA; 7 University of Miami (UM) Clinical and Translational Science Institute, Miami, FL, USA

**Keywords:** COVID, community engagement, CTSA, health disparities

## Abstract

**Introduction:**

Prior to the COVID pandemic, many CTSAs employed face-to-face interactions to conduct most of their community engagement (CE) activities. During the COVID pandemic, such engagement had to be curtailed and alternatives needed to be formulated. In addition, Community Engaged Research (CEnR) teams refocused their efforts to address this public health crisis.

**Methods:**

To obtain a general understanding of how CTSAs have conducted CE and CEnR during the COVID pandemic, we invited seven CTSA CE leaders to provide brief field reports of their activities during the pandemic. This included how their approaches to CE and CEnR were modified during the COVID-19 pandemic and key lessons learned.

**Results:**

We found that despite numerous challenges, all seven CTSAs CE cores were able to successfully carry out CE and CEnR. We also found that the fundamental principles of meaningful and authentic stakeholder engagement were of paramount importance during the pandemic. Through virtual approaches, all sites had considerable success in maintaining CE in during the COVID pandemic. They also leveraged existing bi-directional community partnerships to carry out meaningful and impactful research. This included both new COVID CEnR and also innovative approaches to sustain prior non-COVID research.

**Conclusions:**

These findings suggest that academic-community partnerships must be fostered and sustained over the many years so that when such crises emerge, all partners can build on existing trust and mutual respect. The lessons learned and the new tools and approaches developed would be key in addressing any such future public health emergencies.

## Introduction

Community engagement (CE) is a crucial feature of successful translational research [1,2]. Accordingly, since inception, each CTSA has been required to have a core dedicated to ensuring that communities are meaningfully engaged in translational research and to also facilitate the bidirectional flow of expertise. The similarities and differences in CE strategies across CTSAs have been previously reviewed [3,4]. In most instances, a major component of CE in all CTSAs has involved face-to-face interactions. However, in response to the COVID pandemic (for rest of this article, the pandemic is simply referred to as “COVID”), each CTSA had to develop CE strategies that followed social distancing guidelines with limited in-person interactions. In addition, COVID disproportionately affected our nation’s most vulnerable populations including racial and ethnic minorities [5,6]. Improving health outcomes and reducing disparities in such communities has also been a long-standing goal of the CE components of the CTSA program. Thus, CE investigators had to urgently pivot their work to develop CE initiatives and Community Engaged Research (CEnR) projects aimed at addressing this unprecedented public health crisis. In this manuscript, we provide a series of field reports describing how seven CTSAs conducted CE and CEnR during the first several months of COVID.

## Methods

For this special JCTS COVID-19 theme issue, seven CTSA CE leaders were invited to describe some of their activities during the COVID pandemic. These seven leaders were a representative sample covering varied geographies, ethnicities, races, and identities. They were asked to provide brief field reports describing how practices were altered, redefined, modified, and/or streamlined to address the challenges and exigencies of COVID. Rather than a comprehensive review of all local initiatives, each leader was asked to provide selected examples with some being similar to other CTSAs and some unique to their Hub. They were also asked to reflect on key lessons learned and which practices should be continued beyond the pandemic and which (if any) should not.

## Results

### University of Miami (UM) Clinical and Translational Science Institute

Prior to COVID, most CE activities such as Community Advisory Board (CAB) meetings and CEnR project meetings involving academic and community partners were held in-person. Then, on March 13, UM paused all noncritical research. Research that could be done remotely and which did not involve direct human contact could proceed. Overnight, investigators needed to find novel and creative solutions to sustain CEnR. Most teams turned to community partners and staff to formulate joint solutions. Many partners had limited experience with video conferencing and initially most meetings were phone based. The learning curve for video conferencing was a challenge but ultimately teams found ways to successfully transition.

Community input was essential in making key CEnR project decisions. One example was a community-based participatory research study involving home based and/or point-of-care (POC) preventive health screenings [7]. In the study Community Health Workers (CHWs) did home visits to help participants conduct these tests. Our community partners developed creative ways for our team of CHWs to conduct these tests without face-to-face interactions. For example, self-sampling for Human Papilloma Virus was done by dropping off the kits and providing instructions by video chat (e.g., WhatsAppp). HIV oral tests were left in the front door for CHWs to immediately process. Using these approaches, nearly all persons were screened without direct contact. Community partners also helped us formulate novel ways to provide participant remuneration. Some participants chose e-gift cards. For others, CHWs dropped gift cards at homes and took pictures from their cars of the participant picking up the card as proof of delivery. Surprisingly, retention rates also improved. At 6 months, all participants had to complete a 20-minute survey administered in their home by a member of the research team. Pre-COVID, our retention rate was 73% (395/542), which is typical of our studies with low-income immigrant communities. When we changed to phone-based surveys, our retention increased to 90% (191/212). However, it is unclear if this improved retention rate was due to simply switching from face-to-face to phone or that COVID restrictions made it was much easier to reach participants at home whom may also have had more free time to talk by phone.

Another project where we had to rethink CEnR was the *All of Us Research Program* [8]. After the study was paused, the team piloted digital outreach and enrollment strategies. Through phone and video chat outreach, staff helped participants complete initial surveys online. Once the pause is lifted, participants will only need to undergo physical measurements and phlebotomy to complete their full enrollment. Lastly, many of our CE research staff were also redeployed to COVID-related research. Researchers helped conduct county-wide community-based public health surveillance involving COVID antibody testing in 4,000 persons [9]. Our staff have also played a major role in COVID vaccine trials. We enrolled over 700 persons for two such trials of which 60% were Latinx and 17% Black. We are also leading a statewide initiative under the NIH’s Community Engagement Alliance (CEAL) Against COVID Disparities [10].

### University of Kentucky Center for Clinical and Translational Science (UK CCTS)

In response to COVID pandemic, the CCTS has modified both the focus of and approach to CE. Guided by our community partners, we have channeled efforts to meet basic health and social needs. As example, based on the community-identified need to provide COVID-related information, we collaborated with our partners to develop English and Spanish websites [11]. The websites serve as repositories of educational materials, related local and national resources, and up-to-date information on critical services such as locations and hours for sites offering free virus testing, food banks, and similar services. Partnering with the UK Athletics Department, we developed Public Service Announcements supporting appropriate use of masks and handwashing among young people of color [12]. In partnership with the community, we mobilized a coalition of local leaders to support efforts to decrease the impact of COVID on the Kentucky Latinx community. Responsive to community input, the coalition is conducting campaigns to educate the community about COVID protective activities, including vaccination.

To ensure continuity, we modified approaches to several CE programs. Among these is our Seed Grants through which Appalachian organizations receive funding to implement health programs. In partnership with grant recipients, funds have been directed toward strengthening community safety nets for those most adversely impacted. We also converted the Community Leadership Institute of Kentucky (CLIK) training program from an in-person to virtual approach. There was consensus among community partners that CLIK, designed to empower community leaders to reduce health disparities through implementation of evidence-based health programs [13], has never been more critical. We also transitioned to virtual meetings with the CCTS Community Champions Cabinet. Comprised of representatives from health, educational, and social organizations throughout Appalachian Kentucky, input from the Cabinet continues to be pivotal to informing our current and future CE efforts.

Concurrently with these activities, we have supported CEnR projects directed toward lessening the pandemic’s impact. We became a critical partner with the COVID Unified Research Experts (CURE) Alliance, collaborating on and reviewing pilot proposals, and facilitating researchers’ engagement with communities. This initiative has resulted in 32 funded studies ranging from the effectiveness of tele-health services to optimization of point-of-care COVID testing to understanding the impact of COVID on diverse populations. In response to the regional impact of the health crisis, we collaborated with another Appalachian Translational Research Network (ATRN) CTSA, Clinical and Translational Sciences Institute, university and community organization members to publish a special edition of the ATRN Newsletter to disseminate COVID-related information [14]. The ATRN is also conducting a survey to improve our understanding of the impact of COVID on our Appalachian communities to support a coordinated multistate response.

### Michigan Institute for Clinical and Health Research (MICHR), University of Michigan

MICHR’s CE Program had already developed and enhanced capacity, resources, and infrastructure to support equitable CEnR partnerships. Thus, we were well positioned to partner early with community members and organizations to get through the pandemic together.

We have learned from our community partners, that in times of crisis and hardship, it is critical to initially come together over immediate needs and secondarily over research—even in our capacity as a CTSA. In April, our Director met with our Community Advisory Board, which advises MICHR Leadership. Our community partners provided feedback on ways we could address immediate needs of communities throughout the state. Additionally, our CE Program created an online survey to ask 1) What are the top three issues facing your community during the pandemic? 2) What questions, if any, do you and your community have that can be translated into research to better understand and address community needs? 3) What questions do you have for U-M leaders during the coronavirus response? 4) What type of support can MICHR offer you in response to the outbreak? 5) What type of information is most valuable to you at this time? and 6) What communications channels do you most frequently use for COVID updates? Results of this ongoing survey allow us to continue to meet both immediate and long-term needs using trusted channels within the community. We partnered with the U-M College of Pharmacy and MICHR leadership to deliver 181 gallons of hand sanitizer to seven counties across the state and helped distribute PPE, food, and personal products (menstrual hygiene, incontinence, baby, etc.) to communities in need.

Another major focus during COVID has been helping bridging the literacy, language, and culture gap. Our community partners reported that educational materials were not inclusive or accessible from a literacy or cultural perspective. We developed flyers tailored to literacy level (2^nd^ to 3^rd^ grade reading level), language spoken (English, Spanish, Arabic, Japanese, and Chinese), and age group (youth and older adults). These have been distributed digitally to >100,000 persons and in print to 17,000 people across the state. In addition, there has been an alarming decline in vaccination rates during COVID across all age groups. While a decline in vaccinations places the health of all communities at risk, communities of color, immigrant populations, and lower income individuals are at greater risk due to social determinants of health and decreased access to and uptake of vaccines. In response to these concerns and in anticipation of vaccine clinical trials, we also developed informational flyers on vaccines, vaccine safety, and vaccine trials that have been distributed via a number of platforms across our network.

Throughout the pandemic, we also needed to pivot the infrastructure we provide for CE research and transitioned to a largely virtual platform. Pivoting our CEnR Infrastructure resulted in increased engagement with more rural areas and communities of color around the state. MICHR also hosts an annual statewide retreat, Building Bridges, Breaking Barriers in Translational Research (BBBB). This year, the event, which provides networking opportunities to build CEnR partnerships and bidirectional education, was offered virtually. Partners (*n* = 112) representing community organizational stakeholders, including community-based nonprofits, health systems, advocacy groups, faith-based institutions, universities, and government agencies, were in attendance. We were able to share best practices for CEnR during the pandemic in a symposium entitled “Trust, Trials, and Treatment.”

Similar to Miami, along with our network of state-wide partners, we are also participating in the NIH’s CEAL program. Our initiative leverages our long-term trusted relationships in the community and the academy toward understanding factors that contribute to the disproportionate burden of COVID in underserved communities. We are also working to identify and implement effective, community-focused strategies to enhance education, awareness, access, and inclusion of underserved communities in research designed to advance the prevention of COVID and reduce disparities across the state.

### The Rockefeller University (RU) Center for Clinical and Translational Science (CCTS)

Unique among CTSAs, the CCTS is based in a research-only institution, which does not deliver medical care. Our CE centers on the cultivation of relationships with new communities and development or conduct of CEnR protocols with RU investigators and community partners. We develop projects that align and pair mechanistic and community aims as part of our Full Spectrum Translational Research model. We also work closely with the Clinical Directors Network- a Practice-Based-Research-Network.

As the pandemic arrived, we initiated various new CeNR projects. One of our first major COVID outreach projects involved one of the first affected communities, a small religious community located in New Rochelle, NY. Our outreach was facilitated by a trusted member of the community who had a strong connection to the research laboratory and acted as the go-between with the community, the researchers, and CCTS. Through a series of Zoom calls, they advised the CCTS team on approach, text, and images for outreach materials, and then distributed them to the community. They also conveyed to us community feedback. The research team, previously trained in our Full Spectrum Translational Town Hall Meeting model, conducted outreach to the affected community through a Zoom town hall, coled with the community member. It was attended by approximately 70 community members. Ultimately, this CE facilitated a CEnR protocol that enrolled 145 individuals convalescing from the initial COVID outbreak, resulting in novel insights into the antibody response to the virus [15]. Since then, the research team and community have sustained the relationship for a study on the natural history of COVID immune response [16].

Maintaining ongoing CEnR through virtual engagement was also prioritized. At the outset of the pandemic, RU research was restricted to COVID-related research. In response, CE researchers had to find solutions to sustain non-COVID CEnR. One example was a DHHS-funded study of dietary and behavioral intervention to lower blood pressure among community-living seniors. The intervention, delivered through congregate meals, included educational sessions, in-person collection of cardiovascular measures in a community-based setting, ongoing social support for behavioral modifications, and periodic downloading of home self-monitoring of blood pressure. In response to pandemic, we moved all activities to remote platforms. Monthly project team meetings and quarterly Advisory Committee meetings were conducted virtually over Zoom. As all members lead senior services organizations, they were adept with the technology and this was a smooth transition.

We also needed to move interactions with community-living senior study participants to remote platforms. This required considerable effort and resulted in variable success. For example, in-person tablet-based survey administration was transitioned to remote capture. Seniors varied in their ability to use technology even when they had a smart phone. Some could download links and complete electronic surveys on their own. Others required telephone or zoom coaching. Also, some required administration of the survey over the telephone and others wanted it by mail. Another example was blood pressure monitoring, which now needed to be done remotely. Telephone coaching was done to help seniors upload their data. Some seniors were able to complete remote monitoring while others could not and preferred to read aloud or take pictures and send several weeks’ worth of measurements at once. In addition, participants received support for blood pressure self-efficacy through in-person nutritional education sessions that were previously well attended. Due to social distancing, these were converted to Zoom webinars. Seniors were mailed links with written instructions and telephone coaching was also provided. However, few seniors logged into the sessions.

Another major focus of the CCTS was maintaining training activities. This year, our Clinical Scholars orientation to community engagement was conducted virtually. In the Fall, the curriculum for our certificate course on community engaged research was provided remotely, including interactive learning activities conducted in virtual breakout rooms. In addition, we have sought to stir additional interest in CEnR among investigators through virtual Town Halls. These were simpler and much less expensive to conduct remotely than prior in-person events.

### North Carolina Translational and Clinical Sciences (NCTraCS) Institute, University of North Carolina (UNC)

At UNC, physical distancing measures and the rapid change to a remote work environment became an immediate challenge to CE investigators conducting traditional face-to-face engagement including qualitative research. Thus, we recognized a need to provide CE investigators with guidance on doing research during the pandemic. In response, our CE program developed a comprehensive guide for stakeholder engagement during COVID. To date, it has been downloaded over 2,400 times (Online Appendix A). Another guide for conducting remote qualitative data collection has had over two dozen downloads (Online Appendix B). We have also conducted 45 consultations for CEnR projects related to COVID and provided direct research support to four of these studies. Many of these consultations have focused on transitioning to remote engagement and data collection methods.

We have also partnered to support CEnR on two large federal awards. Similar to Miami and Michigan, we also led the state response to the NHLBI CEAL initiative and will convene relevant stakeholders to develop recommendations on how to disseminate high-priority COVID-19 messaging across NC. Given the drastic rise in COVID within local Latinx communities, our MURAL (MULtilingual Research Advancement for heaLth) service pivoted its efforts. Activities focused on providing quick turnaround guidance and Spanish-language support to engage the Latinx community in relevant research including treatment and vaccine trials. This included the development of Spanish language, culturally adapted study materials, and bilingual support for recruitment and informed consent. One example was an observational study of COVID positive patients to identify risk factors for severe disease. Finally, our faculty and staff are supporting a PCORI-funded COVID Engagement Award through the conduct of qualitative research in patients with inflammatory bowel disease.

We are also leading a project to develop effective, sustainable strategies for promoting rapid, remote risk communication strategies for COVID, and future public health emergencies. The project seeks to leverage the power of social connections within rural, Black faith communities. Specifically, it will test existing remote strategies for promoting the World Health Organization’s Risk Communication and Community Engagement Guidelines [17] and develop new approaches that explicitly utilize the rural Black faith communities’ existing social connections. It is anticipated that study findings will provide a model for rural Black populations seeking to achieve equitable access to urgent, culturally sensitive public health information.

Lastly, in response to North Carolina’s COVID Recovery Act [18], UNC is developing a COVID Legislative Dashboard as a comprehensive resource for testing, screening, and surveillance. Our team is overseeing the engagement arm of the project to ensure the public-facing dashboard is informed by community needs and priorities. To date, we have distributed a Qualtrics survey to individuals, agencies, and organizations to better assess their and their constituent communities’ data-related needs and preferences for dashboard content and usability. We have also conducted focus groups with county-specific working groups and one group of local health department leaders. Preferences were voiced for digestible, easy-to-understand data that is disseminated widely, through media, and that can be compared across counties, as well as data related to school-aged children.

### University of Utah Center for Clinical and Translational Science

The catchment area for our hub includes six states in the Intermountain West. Thus, even prior to COVID, some community partners in more distant locations were participating in CE activities through technologies such as Skype, FaceTime, and telephone. When the pandemic restrictions were instituted, we moved all of our engagement sessions to Zoom. These included focus groups, engagement studios, Community Advisory Boards (CABs), and interviews. This transition presented us not only with some challenges but also with some unexpected opportunities.

With respect to virtual platforms, we found that even individuals who had little familiarity Zoom were able to successfully participate after receiving detailed step-by-step instructions. Through smartphones most partners were able to effectively engage in activities requiring participation via video or voice. However, the technology was not adequate for CE activities or CEnR projects in which participants needed to view slides or multimedia pieces, respond to polls, or click on links in the chat for surveys. To address this, projects had to purchase equipment such as iPads or Amazon Fire tablets to give to partners and/or study participants. Sometimes, they also purchased cellular plans for those who do not have home Wi-Fi. A second technological challenge was interpretation. For in-person sessions, we had used simultaneous interpretation into headsets. With Zoom, interpretation takes place over a separate phone call.

At the same time, we have seen some positive impacts. Individuals from distant locations who used to connect remotely for in-person meetings, now report feeling more part of the discussion since everyone is engaged virtually. In addition, before COVID, we conducted much of our recruitment in person. Now, we use social media (Facebook, Twitter) to contact appropriate organizations and ask those with newsletters to share our recruitment fliers, and send fliers to past participants with the request to share the opportunities with family and friends. Further, Zoom also allows us to recruit nationwide for projects with narrow inclusion criteria, thus supporting engagement for a broader range of research.

Feedback from investigators and community members has been positive. While investigators express pros and cons to virtual versus in-person engagement, most feel that the quality of data is the same for both. One investigator noted that virtual breakout rooms resulted in three times more in-depth and useful information than they had received during whole-group, in-person meetings. Yet, most also miss the informal community-building interactions that take place before and after in-person sessions. Community members expressed similar opinions. For example, this summer we held two, 2-hour trainings for leaders from five diverse communities using Zoom. One session focused on recruiting and preparing participants for engagement sessions and the other addressed cofacilitating virtual engagement sessions. In interviews after these experiences, the community leaders reported specific skills and techniques they had learned and subsequently used as well as an increased comfort with cofacilitating. While they felt the virtual training was very helpful, they would have preferred in-person sessions.

We also feel it is critically important to celebrate and support the creativity of our community partners in developing ways to connect with their members during the pandemic. Thus, we continue to support activities such as the Urban Indian Center of Salt Lake’s health fair. This year it was conducted as a “drive-thru” event. A guide walked along with attendee vehicles as they drove past each table, providing key health information on that topic via phone; participants received a bag of materials with additional information at the end. Another partner, the National Tongan American Society, held several drive-through events for voter registration and census completion. Partners and attendees have expressed preference, when possible, for resuming these as in-person events. However, all are very happy to see the efforts being made to engage and support everyone during the pandemic.

### Oregon Clinical and Translational Research Institute, Oregon Health & Science University

A major CE strategy of our CTSA is through our statewide network of Community Research Liaisons (CRLs). Pre-COVID much of their work was carried out by in-person meetings. Thus, some of the greatest potential workflow impacts of COVID were on our CRLs. However, recognizing their critical role in addressing this public health crisis, our CRLs quickly rose to this challenge. Instead of stopping their work, they immediately repositioned themselves to address the pandemic. As a team, the CRLs identified tele-mental health as an important area of need during this time. In response, they conducted interviews with behavioral health professionals from all five liaison regions to learn how they could support communities in regard to this topic. With the information gathered from the interviews, a summer research intern developed a tele-mental health resource list including topics such as national and regional trainings, reimbursement and billing protocols, and evidence and research on tele-mental health.

In the southern region, the CRL worked with local stakeholders to gather and post resources on their local website. This group was phenomenal in bringing community resources where needed. They also contacted local businesses and agencies to “open” access to their Wi-Fi networks so people could logon in parking lots and coordinated kid’s brown bag lunch pick-up. In another community (the Gorge), the CRL co-hosted a weekly series of Rapid Response Zoom calls where she organized panelists to present information on current services, resources, and information related to the weekly topics, which included food security, housing, and finances. Information from these calls was added to the community resource list she created. In the north coast, the CRL worked with their local stakeholder group to implement a Rapid Response Task Force and a Rapid Response Communication Plan. The key objectives were to connect, mobilize, and incubate. They are now facilitating monthly round tables on different topics related to COVID with 3–5 panelists sharing information and also sending out weekly communication on one topic, issue, or concern, including links to resources.

In addition, a key activity for our community program is in supporting “in reach” to our investigators. We support investigators in bringing the voice of the community to their work, ensuring greater relevance and receptivity of research. One approach we had started implementing is the Community Engagement Studio (CES) to facilitate open and meaningful interchange between a selected community experts and researchers. An important aspect of the CES is in person meetings. During COVID, the need for community voice in research became acutely apparent and for our community program, that meant working to continue to make our CES model possible and effective virtually while maintaining fidelity to the CES model. The CE team explored video conferencing options, use of different methods to encourage open engagement and initiated the first virtual CES in November. Reviews from both community experts and researchers were very positive. Given the large geographic area we support (state of Oregon), we are excited to continue this virtual CES approach even after COVID to increase our ability to reach to community experts throughout the state.

## Discussion

The above field reports provide concrete granular examples of how seven CTSA CE cores modified CE and CeNR practices during COVID. The profiles of these seven institutions highlight the diversity, strength, and resilience of CTSA CE cores and their community partners during this pandemic and also the key roles that such cores can play in quickly responding to similar crises in the future. Despite the challenges of COVID, all had considerable success in maintaining CE and CEnR.

Consistent with prior studies [19], our findings again reinforce the importance and centrality of CE in addressing COVID. We showed how these cores were able to successfully partner with, learn from, and support community interests and priorities to mitigate the disastrous impacts of COVID in some of our most vulnerable groups. In many ways, our CE experiences parallel those of other public health programs working to address COVID [20]. The main distinction is that in addition to maintaining critical CE, all sites were also able to continue to serve as important CEnR research hubs. All were able to promote new COVID CEnR while also being able to find innovative approaches to sustain prior ongoing non-COVID research.

Collectively, we have learned a number of important lessons highlighting the critical role of CTSA CE leadership and infrastructure during such a pandemic. In Table 1, we provide a brief summary on these key lessons and future directions. One overriding theme is that during COVID, our CE infrastructures needed to pivot toward meeting the needs of the community which in the face of a pandemic may not primarily be research related. Indeed, in most of the cited case examples, our research platforms were able to successfully pivot to meet compelling community needs—while still being effective at supporting research. We believe such lessons can better inform and position CE and CEnR teams to efficiently and effectively respond to future public health emergencies. We conclude that through flexibility and adapting successful approaches, along with our community partners, we have not just survived COVID, but have identified additional opportunities to thrive and meet similar future challenges.

**Table 1.** Key lessons learned and future directions

Leadership by CTSA CE cores	During the pandemic, all seven CTSAs played a critical lead role in both community engagement and in design and conduct of high-impact CEnR. Ongoing leadership by such cores will be key in quickly responding to similar watershed experiences in the future.
Infrastructure for supporting long-term Engagement	All CTSAs leveraged existing long-term bidirectional community partnerships. These relationships must be fostered and sustained long-term so when such crises suddenly emerge, all partners can build on existing trust and mutual respect
Need for Community engagement in times of crisis	The fundamental principles of meaningful and authentic stakeholder engagement are of paramount importance during a public health crisis. These include continuous check-ins, maintaining rather than withdrawing from engaged relationships, identification and recognition of challenges, recalibration of expectations and timelines and most importantly, prioritizing the core needs of community partners.
Community engagement can be successfully done virtually	Despite long-standing approaches involving face-to-face engagement, we realized if needed it is possible to leverage technology, including video-conferencing and asynchronous communication, to support remote engagement and CEnR. We found many components of CE and CEnR can be done though distance and non-face-to-face approaches. Such virtual approaches will be an important additional tool that we will to continue to include as part of CE and CEnR strategies for our investigators, community partners and study participants.
Virtual engagement does not replace face-to-face interactions	Many academic and community partners miss the personal interactions that are at the heart of our work. Many would like to resume these when safe and possible to do so.

CTSA, Clinical and Translational Science Award Program; CE, Community Engagement; CEnR, Community Engaged research.

## Supplementary material

For supplementary material accompanying this paper visit http://dx.doi.org/10.1017/cts.2021.785. click here to view supplementary material
