# Supplementary material for: Community engagement during COVID: A field report from seven CTSAs
Source: J Clin Transl Sci. 2021 Apr 27;5(1):e104. doi: 10.1017/cts.2021.785 (PMC8185415; doi:10.1017/cts.2021.785)
Supplement: Supplementary file 1 [file ctssup.zip › S2059866121007858sup001.docx]

**Engaged Research Partnerships During COVID-19:**

**Staying True to Principles of Community and Stakeholder Engagement during the COVID-19 Pandemic**

The [Community and Stakeholder Engagement Program](https://tracs.unc.edu/index.php/services/engagement) at the North Carolina Translational and Clinical Sciences (NC TraCS) Institute at UNC-Chapel Hill promotes and facilitates the engagement of communities and stakeholders in research. The COVID-19 pandemic has created unique circumstances and important considerations for community-engaged research. Research projects and partnerships are adjusting to this crisis in a variety of ways, particularly given the challenges of adhering to the principles of community engagement while practicing social and physical distancing. NC TraCS has compiled this document to provide guidance to community and academic stakeholders to help them remotely maintain social connections and navigate partnerships during this challenging time. *As circumstances are rapidly changing, we will continue to update this document as is relevant. The most recent version can be found in the publications toolbar at:* [*tracs.unc.edu/engagement*](https://tracs.unc.edu/index.php/services/engagement)*.*

*For more information about NC TraCS services related to community-engaged research, visit* [*www.tracs.unc.edu/engagement*](http://www.tracs.unc.edu/engagement)*. For local resources related to the COVID-19 pandemic in North Carolina, visit:* [*https://www.ncdhhs.gov/divisions/public-health/covid19*](https://www.ncdhhs.gov/divisions/public-health/covid19) *and* [*https://bit.ly/COVID-NCIDR*](https://bit.ly/COVID-NCIDR)*.*

*We would like to acknowledge the Alliance for Research in Chicagoland Communities (*[*ARCC*](http://www.arcconline.net/)*) for their significant contributions to this document, and for their dedication to supporting their local communities. To learn more about their work and to access other resources related to community-engaged research, visit* [*www.ARCCresources.net*](http://www.arccresources.net/)*.*

Contents

[Supporting Engaged Partnerships 2](#_Toc37937324)

[Adjusting Expectations and Work of Research Partnerships 3](#_Toc37937325)

[Adjusting Research Protocols during COVID-19 4](#_Toc37937326)

[Considering Equity Issues during COVID-19 4](#_Toc37937327)

[Practical Tools for Virtual Engagement 5](#_Toc37937328)

[Tips and Resources for Virtual Meeting Facilitation: 7](#_Toc37937329)

[Leveraging Resources to Support Partners 8](#_Toc37937330)

[The NC TraCS Community and Stakeholder Engagement Program is here to help! 9](#_Toc37937331)

# Supporting Engaged Partnerships

***Note that these suggestions are primarily for previously established partnerships. This will likely be a difficult time to try to begin new relationships unless the work is specifically related to COVID-19.***

- - **Express concern** for the health and safety of your stakeholder partners, their families, and their communities.
  - Do not make assumptions about what people need. **Ask** them directly.
  - **Check in** continuously, not just once or at the beginning of the crisis.
  - **Acknowledge** the rapid changes that are occurring, and the collective goal of finding ways to move forward and troubleshoot together.
  - Possible check-in questions include:
    - How are you doing? Your family? Your community?
    - What information or support are you missing? What has been the most helpful? What is confusing?
    - What are you looking for from your research or community partners right now?
    - What is your capacity to keep working together?
    - *Note: Also share your own answers to these questions.*
  - **Offer support and resources** as available and based on your stakeholders’ expressed needs
  - **Do not pull away from engagement during a crisis.** In the moment, it may feel necessary to make changes to your research project quickly rather than spending added time and effort discussing changes with community partners. However, communities impacted by changes should be engaged in the change process. If this is unable to happen because of time or availability, then all partners should be notified in a timely manner.
  - Consider the balance between maintaining communication and not adding burden with too much outreach. Ask your partners directly about their desired frequency of communication and level of engagement during this time.
  - Consider what **communication methods** will work best in this moment. Ask partners about preferred methods of communication and follow through accordingly.
  - **Acknowledge the importance of mental health and balance**. Partnerships do not have to be as or more productive during the pandemic. There is a benefit to taking time to stop, breathe, and reflect.
  - Offer and receive grace. Be patient and gentle with each other.
  - **Celebrate** each other and continue to focus on assets and strengths.

#

# Adjusting Expectations and Work of Research Partnerships

- - Consider that everyone may have different priorities, availability and bandwidth. This includes community partners, research participants, research faculty, and research staff. Availability may change over time.
  - **Acknowledge that things will be different during the pandemic**. Work will not continue at the same pace. Acknowledge clearly that this is okay.
  - Shared challenges may include:
  - Child care/education responsibilities
  - Family caregiving responsibilities
  - Financial stress
  - Challenging remote work environments (e.g. may not have access to tools, documents, hardcopy assets, etc.)
  - Community partners may face specific challenges, including:
    - Community partners may need to dedicate more time to working with the public and have less time available for working on research related tasks.
    - Community partners may be navigating new protocols for how to conduct their essential services (e.g. transitioning to telework).
  - Researchers may also face specific challenges, including:
    - Clinician-researchers may have increased clinical hours and decreased time for research due to acute demand for healthcare professionals.
    - Research protocols may need to be revised with short notice.
  - Consider whether community partners have access to and comfort using technology (e.g. home computer, internet access, etc.) and other virtual communications resources while working remotely. *(See Section 5 for more tips on virtual engagement)*
  - **Timelines might need to shift** for partnership activities, research activities, dissemination, grant application, and manuscript preparation.
  - **Assess the current phase of the research project and stakeholder partnership**. What’s happening now? What is due to happen in next 6-12 months? What events need to be canceled or postponed? What can be done virtually? What are ways that burdens of work can be reduced, postponed or relieved?
  - **Check with funders**. They may have changed grant deadlines or requirements or be able to change them upon request.
  - Ensure any changes in timelines for proposal submission/review or work deadlines are communicated to all partners.
  - Consider what it means to build and sustain a team even while social distancing.
    - **Resource:** [Building Trust While Working Remotely](https://www.theengineroom.org/building-trust-while-working-remotely/) by The Engine Room

# Adjusting Research Protocols during COVID-19

- - Changes to research protocols may occur to protect the health and safety of research participants and as mandated by university institutional review boards (IRBs). Ensure these changes are communicated to all partners. Consider whether there should be an **engaged conversation between community and academic partners about implications of these changes in the research protocol** on community research partners and research participants. Questions to consider include:
    - - What does it mean for research participants to stop or delay participation in a research study or transition to virtual participation?
      - Were there benefits or resources that research participants had access to that will now be unavailable to them?
      - Should messages related to COVID-19 be incorporated into communications with research participants? For example, if a research project engages people with asthma, should information or resources related to increased risk or protections be incorporated?
      - Should additional questions or data collection about the impact of COVID-19 (health, economic or other) be added to research data collected?
      - Are there new research questions related to COVID-19 that should be considered? Many funders are offering grant funding related to COVID-19 research questions and community needs.
  - University research offices’ operations may be impacted. This may result in changed or delayed process for payment to research staff, Community Advisory Board members, community partners, vendors, etc. Be sure to communicate these changes with stakeholder partners.
  - **Resource**: UNC’s Office of Research Development is maintaining a list of relevant funding opportunities here: <https://research.unc.edu/research-development/finding-funding/covid-19/>

# Considering Equity Issues during COVID-19

- Many are concerned about inequities that COVID-19 may exacerbate. It is likely that health and socioeconomic disparities pre-COVID-19 will be magnified and create other ripple effects that will also negatively impact communities experiencing inequities. A few articles on this topic include:
  - [COVID-19 highlights health disparities facing African-Americans](https://www.insightnews.com/news/covid--highlights-health-disparities-facing-african-americans/article_277d58f0-72f9-11ea-880c-03e80a8b6b53.html)
  - [Coronavirus Compounds Inequality and Endangers Communities of Color](https://www.americanprogress.org/issues/race/news/2020/03/27/482337/coronavirus-compounds-inequality-endangers-communities-color/)
  - [Health issues for blacks, Latinos and Native Americans may cause coronavirus to ravage communities](https://www.usatoday.com/story/news/nation/2020/03/30/coronavirus-cases-could-soar-blacks-latinos-and-native-americans/2917493001/)
  - **Resource:** Racial Equity Tools has a number of [resources focused on addressing COVID-19 with a racial equity lens](https://www.racialequitytools.org/fundamentals/resource-lists/resources-addressing-covid-19-with-racial-equity-lens).
  - **Acknowledge inequities in the impact of COVID-19**. This includes direct health implications, as well as economic and other ripple effects.
  - Acknowledge that communities with high hardship and low resources may face additional challenges.
  - Ensure immigrants or other limited or non-English proficiency communities with whom you are engaging have culturally, linguistically appropriate information. For stakeholders that speak a primary language other than English:
    - - Obtain professional translations of materials and have bilingual staff review these translations.
      - Avoid asking bilingual staff to translate materials unless they are certified translators, as unprofessional translations will reduce the accuracy of the message.
      - Proactively build relationships with professional translators in your area who are native speakers of the primary languages of your stakeholders.
      - Consult with your financial managers to ensure that local translators can complete vendor applications before their services are needed so that they will be available on short notice to translate announcements and current events.
      - Consider having audio recordings of translated announcements that can be made available via cell phones, to avoid literacy barriers.
  - **Speak up against racism and xenophobia**.
  - **Do not pull away from equity during a crisis**. Now is the time to focus on and center equity. It is especially important during times of pressure and emergency. Partnerships must think about the means as well as end results.
  - Consider equity impacts of any changes in research protocol or at institutions. Who will be burdened? Who will be advantaged? How can you help mitigate these effects? For example:
    - - If you are cancelling events or activities, can you still pay vendors, speakers?
      - If you are stopping or postponing grant activities, can you continue to pay hourly employees?
  - Advocate for institutional and government action.
    - - **Resource**: [Demands from Grassroots Organizers Concerning COVID-19](https://transformativespaces.org/2020/03/04/demands-from-grassroots-organizers-concerning-covid-19/)
  - *More suggestions for action are included in Section 6.*

# Practical Tools for Virtual Engagement

- - Your partnership may need to **build capacity and comfort to communicate and engage virtually**. Virtual engagement may include communication by phone, text, email, phone or video conference calls and webinars. Any technology that resonates with your stakeholders and allows for researcher/stakeholder interactions can be harnessed to assist with engagement.
  - Examples of engagement modes and ways they might be utilized include:
    - Phone
      - - One-on-one calls between researchers and stakeholders
        - Group conference calls
    - Snail mail
      - - Mailing out study materials for review before a phone call
    - Email
      - - Emailing study materials for review
        - “Reply all” group conversations
    - Online file sharing
      - - Sharing materials via OneDrive or Dropbox
        - Allowing stakeholders to edit or comment on a Google document
    - Social media
      - - Having discussions via a closed Facebook group
        - Videoconference platforms
    - Hosting online meetings with stakeholders
  - Ensure that **new** **modes of engagement are feasible and acceptable** to stakeholders
    - - Let your stakeholders be your guide! Ask for their preferred way to engage remotely.
      - Be sensitive to barriers stakeholders may face in engagement using a particular method (e.g., lack Internet access)
      - Whenever possible, offer multiple ways to engage. For example, you might provide an option to call in to an online meeting via phone, and send materials out in advance via snail mail.
      - Ask for stakeholder feedback throughout the project and work to address any issues hindering their engagement.
  - If using videoconferencing or other online platforms:
    - Provide participants with necessary technological tutorials or technical support in advance, including written instructions for utilizing the technology.
    - Understand that stakeholders’ technological literacy will vary, and be willing and available to answer questions.
    - Have a champion from the targeted community work with you to acclimate stakeholders to the technology used.
    - When possible, have a lower-tech option for engaging, such as the option to call in to a videoconference via phone
  - Many videoconference platforms exist, such as Zoom, WebEx, Skype for Business, and GoToMeetings. Consider the specific features of each when deciding which platform best meets your partners’ needs. If you will be sharing or discussing study data on a videoconference platform, **consult with your IRB and IT consultants to ensure your chosen platform adheres to HIPAA and data security requirements.**
  - This document outlines features available via Zoom, as it is a videoconference platform widely available at UNC Chapel Hill: <https://software.sites.unc.edu/zoom/>. However, the recommendations below can be applied broadly, as many of these features are available with other videoconference software.
    - - - Information about data types permitted in Zoom at UNC Chapel Hill and how to request a HIPAA enabled Zoom account is available at <https://safecomputing.unc.edu/it-tool/zoom/> and <https://safecomputing.unc.edu/2020/03/updates-to-zoom-re-sensitive-data/>.
        - UNC School of Medicine faculty and staff also have access to WebEx: <https://www.med.unc.edu/it/services/video-conferencing/>
        - Guidance from the UNC Office of Clinical Trials (4/6/20) notes that the UNC School of Medicine WebEx account is HIPAA compliant, but we recommend consulting with IRB / your IT consultant before beginning a project.
  - Take steps to enhance security of videoconference sessions, such as providing participants with a password for joining the call.
    - - Recently there have been incidents of cyber hacking and attacks during Zoom calls. In light of these issues, School of Medicine faculty/staff can consider using WebEx for data collection. If using Zoom, steps study teams can take to enhance security include:
        - Making meetings private
        - Waiting until just before the session to share meeting details with participants
        - Sending meeting links and information directly to participants (not posting online)
        - Using the Waiting Room feature, where the host allows admits participants to the session: <https://support.zoom.us/hc/en-us/articles/115000332726-Waiting-Room>
        - Requiring a password to enter the session: <https://support.zoom.us/hc/en-us/articles/360033559832-Meeting-and-Webinar-Passwords->
        - Note that if attacks occur, the host can remove the caller from the session. Report any issues related to hacking or attacks during Zoom calls to UNC ITS (919-962-HELP)

## Tips and Resources for Virtual Meeting Facilitation

- - Identify roles for facilitating the session (e.g., facilitator, notetaker, technical support person). Consider having stakeholders take on some or all of these roles.
  - At the beginning of the session:
    - Offer a brief tutorial on how to use the online platform
    - Go over ground rules, including how to take turns speaking (e.g., raising a (virtual) hand and waiting to be unmuted)
  - Pay attention to who is speaking and provide opportunities for all participants to share. If some participants are more active than others, ask, “Would someone who hasn’t shared yet like to speak?”
  - Consider using videoconference tools to support your discussion. Examples of these tools in Zoom and ideas for their use include:
    - Whiteboard -- <https://support.zoom.us/hc/en-us/articles/205677665-Sharing-a-whiteboard>
      - Summarizing: record responses on the white board and ask stakeholders to confirm or discuss
      - Brainstorming: invite stakeholders to write directly on a shared screen
    - Screen share - <https://support.zoom.us/hc/en-us/articles/201362153-Sharing-your-screen>
      - Share materials and ask stakeholders to react/provide feedback
        - Demo a website or tool and ask stakeholders to provide feedback
      - Poll - <https://support.zoom.us/hc/en-us/articles/213756303-Polling-for-Meetings>
        - Conduct a vote
        - Seek initial reactions to a question
      - Meeting reactions - <https://support.zoom.us/hc/en-us/articles/360038311212-Meeting-reactions>
        - Ask meeting participants to indicate agreement or disagreement using the thumbs up/ thumbs down feature
      - Chat - <https://support.zoom.us/hc/en-us/articles/203650445-In-Meeting-Chat>
        - Some meeting participants may prefer to engage by writing in the chat box vs. speaking
        - Troubleshoot individual technical issues via chat.
      - Breakout rooms - <https://support.zoom.us/hc/en-us/articles/206476093-Getting-Started-with-Breakout-Rooms>
        - Have committees split off into breakout rooms
        - Have small groups converse and then report back to the group
  - **Other Resources:**
    - [Online Meeting Resources Toolkit for Facilitators During Coronavirus Pandemic](https://docs.google.com/document/d/1NyrEU7n6IUl5rgGiflx_dK8CrdoB2bwyyl9XG-H7iw8/edit?pli=1&amp;heading=h.jb9co2l7jt1p)
    - [Virtual Meeting Check-Ins and Icebreakers during a Pandemic](http://www.bethkanter.org/pandemic-icebreakers/)
    - [Check in Questions](https://www.storybasedstrategy.org/blog-full/2019/9/18/checking-in-to-prevent-checking-out) from the Center for Story-based Strategy.
    - [Virtual Ice Breakers Help Remote Teams Break the Ice](https://www.google.com/search?rlz=1C1GCEU_enUS838US838&amp;biw=800&amp;bih=539&amp;ei=-AKEXvb-JaaP9PwP1bqM8A4&amp;q=Virtual%2BIcebreakers%2BHelp%2BRemote%2BTeams%2BBreak%2Bthe%2BIce&amp;oq=Virtual%2BIcebreakers%2BHelp%2BRemote%2BTeams%2BBreak%2Bthe%2BIce&amp;gs_lcp=CgZwc3ktYWIQAzoECAAQR1D3GliDJGC8KGgAcAF4AIABN4gBX5IBATKYAQCgAQGqAQdnd3Mtd2l6&amp;sclient=psy-ab&amp;ved=0ahUKEwj22tiyncboAhWmB50JHVUdA-4Q4dUDCAs&amp;uact=5)
    - [Online Interaction Tools to Engage Your Audience](http://www.bethkanter.org/online-interaction-tools/)
    - [Leading Groups Online Free-e-book](https://www.trainingforchange.org/training_tools/leading-groups-online-book/)
    - [Digital Divide Resources during the COVID-19 pandemic from NTEN](https://www.nten.org/article/digital-divide-resources-during-the-pandemic/)
    - [Everyone On: Low or no-cost internet service](https://www.everyoneon.org/find-offers)
    - 60 days of Free Internet for qualifying households

# Leveraging Resources to Support Partners

- - Research partners may be able to **leverage their institutional resources** to support community partners. For example, research partners may have access to COVID-19 information, data tools, online library or learning resources, or connections to health professionals.
  - Ask community partners how research partners can help with outreach/education about COVID-19 and desired resources for community members.
  - Amplify community messages/request for support
    - Advocate for institutional and government action
    - Write or share op-eds or blogs about the impact of COVID-19 on communities experiencing inequities- not just in national or mass media but local community media. Ask: What are topics that research partners can lend their voice to and shine a light on?
    - Areas for advocacy may include:
      - - Ensuring that COVID-19 testing and clinical care is equitable and resource allocation is fair
        - Providing for the communities who lack basic supplies
        - Highlighting the financial impact that quarantine and shelter-in-place orders have on patients and communities and explore ways to support them
        - Highlighting the needs of the housing-insecure people experiencing homelessness, ‘essential workers’ who are unable to work remotely, and other populations who are at risk for COVID-19
  - **Help partners navigate information**: Help communities to understand and sort through the constantly changing high volume of available COVID-19 information.
  - Be available and willing to answer community questions or connect them with resources or people to answer questions.
    - Host a webinar/Zoom meeting to answer community fielded frequently asked questions. This may be especially helpful for providing more detailed information about specific topics. For example, “what does COVID-19 mean for me if I’m a diabetic?”
  - **Donate/provide financial support**. Consider whether research faculty or institutions can support efforts to support community individuals or organizations.
  - **Donate Personal Protective equipment (PPE)** to share with healthcare providers or community partners.
  - If your research project or partnership is directly engaging people with elevated risks from COVID-19 (e.g. homeless people, elders, immunocompromised people, sex workers, drug users, incarcerated people, or others), how can you help them access medical and risk reduction supplies that will help them survive?
  - **Volunteer**: Many opportunities exist to deliver food to older adults, help teach tech, babysit, etc.
  - **Share skills**. Do partners have expertise in data visualization, epidemiology or other relevant skills to share?

# The NC TraCS Community and Stakeholder Engagement Program is here to help!

- - We offer free consultations and can support you as you navigate remote stakeholder engagement
  - Examples of ways we can assist include:
    - Working with your team to plan remote stakeholder engagement activities
    - Reviewing engagement plans
    - Observing and providing feedback on a remote engagement session
    - Providing tutorials on using videoconference features to assist with stakeholder engagement
    - Assisting in designing technology platform tutorial materials for your population
    - Helping problem solve issues that arise
    - Assisting with facilitating remote engagement sessions (chargeback rate applies)

**Contact us for a consultation** by emailing [engagement.nctracs@unc.edu](mailto:engagement.nctracs@unc.edu) or submitting a request at <https://tracs.unc.edu/consultation>.
