# Supplementary material for: Community engagement during COVID: A field report from seven CTSAs
Source: J Clin Transl Sci. 2021 Apr 27;5(1):e104. doi: 10.1017/cts.2021.785 (PMC8185415; doi:10.1017/cts.2021.785)
Supplement: Supplementary file 1 [file ctssup.zip › S2059866121007858sup002.docx]

**
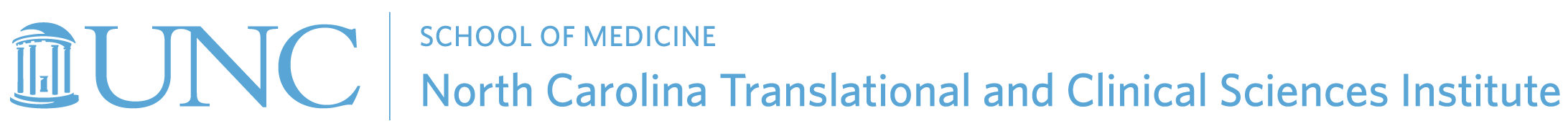
**

**Tips and Tools for Remote Qualitative Data Collection**

Phone and videoconference interviews are common qualitative data collection methods. While less common, focus groups can also be conducted remotely using videoconference platforms. With thoughtful decision making and planning, remote qualitative data collection can be conducted successfully and with scientific rigor.

When conducting remote qualitative data collection, the basic principles of in-person interviewing and focus group facilitation apply. Unique considerations for remote qualitative data collection are discussed below. For questions, additional information, or support, please contact us at [engagement.nctracs@unc.edu](mailto:engagement.nctracs@unc.edu).

*Please note that this is a working document and we are continually seeking to refine these guidelines. If you would like to provide any feedback on the guidelines, please contact us at* [*engagement.nctracs@unc.edu*](mailto:engagement.nctracs@unc.edu)*. You can access the most current version of the guide at* [*https://tracs.unc.edu/index.php/services/engagement/qualitative-research-services*](https://tracs.unc.edu/index.php/services/engagement/qualitative-research-services)*.*

Contents

[Navigating regulatory approvals 1](#_Toc43808615)

[Setting yourself up for technological success 2](#_Toc43808616)

[Preparing participants to engage in remote qualitative research activities 3](#_Toc43808617)

[Conducting phone or videoconference interviews 4](#_Toc43808618)

[Facilitating remote (videoconference) focus groups 5](#_Toc43808619)

[NC TraCS Qualitative Research Specialists are here to help! 6](#_Toc43808620)

[Appendix A: Sample Information Sheet Template 7](#_Toc43808621)

[Appendix B: Sample Verbal Consent Script 8](#_Toc43808622)

[Appendix C: Additional Resources on Remote Qualitative Data Collection 9](#_Toc43808623)

# Navigating regulatory approvals

- Your IRB protocol should note clearly that study procedures are occurring remotely. If your IRB protocol indicates that certain processes will occur in-person, it should be revised to note that these processes will take place via phone or videoconference.
  - Include all necessary study changes in your revision. Common modifications involve those to consent processes, data collection methods (including virtual platforms used), and compensation methods (e.g., e-gift card).
  - *FAQs on UNC IRB modifications due to COVID-19 can be found at* [*https://research.unc.edu/2020/03/10/ohre-irb-covid-19-update/*](https://research.unc.edu/2020/03/10/ohre-irb-covid-19-update/)
- In certain situations, the IRB may allow for verbal consent processes.
  - A verbal consent process may include emailing participants a concise information sheet about the study, including risks and benefits of participating, in advance. The interviewer or focus group facilitator can use a script to obtain consent from the participant before the interview or focus group begins.
    - A sample information sheet template and verbal consent script can be found in Appendices A and B.
  - If you will be audio and/or video recording, participants must consent to being recorded. Your study should determine in advance whether you will allow participants who do not consent to recording to continue participating, and make that clear to participants.
  - Consult with IRB and your IT consultants about audio/video recording options and videoconference platforms to ensure they adhere to HIPAA and data security requirements.

# Setting yourself up for technological success

- Determine the technology you will use for the data collection activity and how you will record the conversation (if applicable). Things to consider when selecting technology include:
  - For interviews, whether a phone call is sufficient, and the added value, if any, of a video call. Consider providing participants with the option to join via phone or video based on their preference.
  - The technological capacity required of participants to complete a phone vs. a video interview.
  - The technological capacity required of participants to engage in a focus group via videoconference, and whether individual phone or video interviews may be better received.
  - Barriers to connecting via videoconference, such as broadband or mobile data coverage and access to a computer or smartphone, and how these barriers may disproportionately affect particular populations who experience health disparities (e.g., rural, low-income populations).
- Many videoconference platforms exist, such as Zoom, WebEx, Skype for Business, and GoToMeetings. Consider the specific features of each when deciding which platform best suits your study’s needs. **Before selecting a platform to use, consult with your IRB and IT consultants to ensure your chosen platform adheres to HIPAA and data security requirements.**
  - *This document outlines features available via Zoom, as it is a videoconference platform widely available at UNC Chapel Hill:* [*https://software.sites.unc.edu/zoom/*](https://software.sites.unc.edu/zoom/)*. However, the recommendations below can be applied broadly, as many of these features are available with other videoconference software.*
    - *Information about data types permitted in Zoom at UNC Chapel Hill and how to request a HIPAA enabled Zoom account is available at* [*https://safecomputing.unc.edu/it-tool/zoom/*](https://safecomputing.unc.edu/it-tool/zoom/) *and* [*https://safecomputing.unc.edu/2020/03/updates-to-zoom-re-sensitive-data/*](https://safecomputing.unc.edu/2020/03/updates-to-zoom-re-sensitive-data/)*.*
  - *UNC School of Medicine faculty and staff also have access to WebEx:* [*https://www.med.unc.edu/it/services/video-conferencing/*](https://www.med.unc.edu/it/services/video-conferencing/)
    - *Guidance from the UNC Office of Clinical Trials (4/6/20) notes that the UNC School of Medicine WebEx account is HIPAA compliant, but we recommend consulting with IRB / your IT consultant before beginning a project.*
- Take steps to enhance security of videoconference sessions, such as provide participants with a password for joining the call.
  - *Recently there have been incidents of cyber hacking and attacks during Zoom calls. Steps study teams can take to enhance security are listed below.*
    - *Making meetings private*
    - *Waiting until just before the session to share meeting details with participants*
    - *Sending meeting links and information directly to participants (not posting online)*
    - *Using the Waiting Room feature, where the host allows admits participants to the session:* [*https://support.zoom.us/hc/en-us/articles/115000332726-Waiting-Room*](https://support.zoom.us/hc/en-us/articles/115000332726-Waiting-Room)
    - *Requiring a password to enter the session:* [*https://support.zoom.us/hc/en-us/articles/360033559832-Meeting-and-Webinar-Passwords-*](https://support.zoom.us/hc/en-us/articles/360033559832-Meeting-and-Webinar-Passwords-)
    - *Note that if attacks occur, the host can remove the caller from the session. At UNC: Report any issues related to hacking or attacks during Zoom calls to UNC ITS (919-962-HELP)*
- Recording options include using speakerphone (in a private area) and recording with a standard digital recorder, recording directly via phone, attaching a recording device to the phone, or using a videoconference platform that allows for recording. **Consult with your IRB and IT consultants to ensure your chosen recording method adheres to HIPAA and data security requirements.**
  - If recording directly on a videoconferencing platform:
    - The recording files may include both an audio and a video file. If you do not want a recording of the participant’s face, you can ask the participants to call in to teleconference (instead of joining online) or turn off cameras before recording.
    - Disable participant recording options for online focus groups to help promote confidentiality.
    - *For more information on setting up and managing local recording via Zoom, see:* [*https://support.zoom.us/hc/en-us/articles/201362473#h_3a53d485-a50f-4f6a-8019-533a0526fe47*](https://support.zoom.us/hc/en-us/articles/201362473#h_3a53d485-a50f-4f6a-8019-533a0526fe47)
- **Practice!** Practice using the technology by having a mock interview or focus group before you begin data collection, and test your recording technology in advance.

# Preparing participants to engage in remote qualitative research activities

- Communicate with participants in advance so they know what to expect when engaging in remote qualitative research activities.
- During the recruitment process:
  - Let participants know if audio/video recording is mandatory (e.g., an online focus group).
  - Ask about participant internet access, if that is a requirement to participate. If possible, have a phone option available for those who do not have stable internet access.
  - Ask participants to join videoconferences from a secure internet connection (e.g., not public Wi-Fi).
  - Ask participants to call from a private location where there will be few interruptions (though be aware that this may be difficult for some participants, such as parents at home with young children).
- If conducting an online focus group, communicate any risks unique to an online environment, such as potential risks to confidentiality (e.g., others in the focus group not having a secure interview connection).
- If using videoconference software, provide participants with tutorials or technical support in advance, including written instructions for joining the videoconference. If possible, allow phone as a backup option. Understand that participants’ technological literacy will vary, and be willing and available to answer questions.
- Let participants know how compensation will take place (e.g., e-gift card emailed to them after the session).

# Conducting phone or videoconference interviews

- Take the call from a private room with no distractions. If using online software, join from a secure Internet connection (e.g., not public Wi-Fi).
- At the beginning of the call:
  - Introduce yourself and the project (this helps with building rapport, particularly when the participant has not met you in-person).
  - Ask participants if it is a good time to talk; suggest that they find a private place to talk.
  - Remind participants that they can end the interview at any time.
- Let the participant know when you will begin recording. State the date and time at the beginning of the recording.
- When you and the participant cannot see each other (e.g., via phone interviews), verbal probing and feedback is especially important.
  - Communicate interest and attention verbally, using techniques such as:
    - Verbal probes (e.g., “mmhmm”, “tell me more”)
    - Reflective listening
    - Thanking the participant for sharing their experiences and perspectives
  - Pause and give participants time to respond. If you both speak at the same time, ask them to continue.
- If there is background noise and/or the participant appears distracted, offer to pause the interview while they attend to the issue.
- Videoconference platforms that allow screen sharing can be very helpful for usability testing interviews. For example, you can ask participants to share their screen as they navigate through a website or tool. Note that there can be a steep learning curve with using some of these features – be prepared to walk participants through the process.
  - *Instructions for accessing these feature in Zoom is available here:* [*https://support.zoom.us/hc/en-us/articles/201362153-Sharing-your-screen*](https://support.zoom.us/hc/en-us/articles/201362153-Sharing-your-screen)*.*

# Facilitating remote (videoconference) focus groups

*Note: The literature notes that online focus groups can be conducted synchronously (with everyone present at the same time) or asynchronously (people present at different times, such as responding to questions and comments via online communication (email, social media, etc.) (Abrams & Gaiser, 2017). This guide focuses on synchronous online focus groups.*

- Join the session from a private room with no distractions. Use a secure Internet connection (e.g., not public Wi-Fi).
- Consider conducting the consenting process individually and in advance, such as during the recruitment call.
- Ensure adequate staffing. This includes:
  - A facilitator
  - A note taker
  - Consider having a third staff person available to provide technical support to participants if needed.
- At the beginning of the session:
  - Offer a brief tutorial on how to use the online platform. Provide instructions for turning participant cameras on or off per their preference.
  - Go over ground rules, including how to take turns speaking.
  - Remind participants about the importance of confidentiality. Ask that they participate from a private room. Ask that they do not record the session.
  - Let participants know when you will begin recording. State the date and time at the beginning of the recording.
- Pay attention to who is speaking and provide opportunities for all participants to share. If some participants are more active than others, ask, “Would someone who hasn’t shared yet like to speak?”
- Teleconferencing software often provides a variety of features for managing participant interactions during a group meeting. Familiarize yourself with these features ahead of time.
  - *Information on managing participants in Zoom can be found at:* [*https://support.zoom.us/hc/en-us/articles/115005759423-Managing-participants-in-a-meeting*](https://support.zoom.us/hc/en-us/articles/115005759423-Managing-participants-in-a-meeting)
- Consider whether using videoconference tools would support your discussion. Note that for participants new to videoconference technology, these tools may be a distraction or barrier to participation.
  - Examples of these tools in Zoom and ideas for their use include:
  - Whiteboard -- <https://support.zoom.us/hc/en-us/articles/205677665-Sharing-a-whiteboard>
    - Summarizing: Record participant responses on the white board and ask them to confirm or discuss.
    - Brainstorming: Ask participants to write responses directly on the whiteboard.
  - Screen share - <https://support.zoom.us/hc/en-us/articles/201362153-Sharing-your-screen>
    - Share materials and ask participants to react/provide feedback.
    - Demo a website or tool and ask participants to provide feedback.
  - Poll - <https://support.zoom.us/hc/en-us/articles/213756303-Polling-for-Meetings>
    - Collect demographic information.
    - Solicit initial reactions to a question and then discuss responses.
  - Meeting reactions - <https://support.zoom.us/hc/en-us/articles/360038311212-Meeting-reactions>
    - Ask participants to indicate agreement or disagreement using the thumbs up/ thumbs down feature.
  - Chat - <https://support.zoom.us/hc/en-us/articles/203650445-In-Meeting-Chat>
    - Ask participants to write responses to a question via chat, followed by discussion. Save the chat thread to refer to after the session.
    - Troubleshoot individual technical issues via chat.

# NC TraCS Qualitative Research Specialists are here to help!

We offer free consultations and are available to help you navigate remote qualitative data collection.

**Examples of ways we can assist include:**

- Working with your team to plan remote data collection activities
- Reviewing interview and focus group guides
- Reviewing participant information sheets
- Participating in and providing feedback on a mock remote interview
- Observing and providing feedback on a mock focus group session
- Providing tutorials on using videoconference features to assist with data collection
- Assisting in designing videoconference platform tutorial materials for your population
- Helping problem solve issues that arise during study implementation
- Conducting remote data collection sessions (chargeback rate applies)

**Contact us for a consultation** by emailing [engagement.nctracs@unc.edu](mailto:engagement.nctracs@unc.edu), or submit a request at <https://tracs.unc.edu/consultation>.

**Learn more about our services at** [**https://tracs.unc.edu/engagement/qualitative-research-services**](https://tracs.unc.edu/engagement/qualitative-research-services)

# Appendix A: Sample Information Sheet Template

**Information Sheet: [Study Name]**

**Who is conducting this research study?**

[List PI name(s), department, and institution.]

**What is this research study about?**

[Provide an overview of the research study, eligibility criteria, and basic activities involved.]

**What will I be asked to do?**

[Outline the activities involved, including the time required to participate, topics covered during interviews/focus groups, and whether audio or video recording is required or optional.]

**Participation is voluntary**

[Sample language*: Participating in this interview is voluntary, and there is no penalty for refusing to participate. If you decide to participate, you can end the interview/leave the focus group at any time.*]

**What are the risks and benefits of participating?**

[Discuss risks and benefits of participating. Outline risks unique to remote data collection, such as video recording, and strategies to protect confidentiality and any limitations (e.g., cannot guarantee other focus group participants will keep information confidential). Discuss who will have access to audio/video recordings and how they will be stored.]

**What are the costs of participating?**

[Outline costs participants may incur, such as costs of a phone call or Internet usage.]

**Will I be compensated for participating?**

[Outline compensation/incentives and how they will be provided to the participant.]

**Who can I contact with questions or concerns?**

[Include contact information for PI and/or study coordinator, as well as IRB information.

Sample IRB information language: *All research on human volunteers is reviewed by a committee that works to protect your rights and welfare. If you have questions or concerns about your rights as a research subject, or if you would like to obtain information or offer input, you may contact the Institutional Review Board at [phone] or by email at [email address].* *The IRB number for this study is [number]].*

# Appendix B: Sample Verbal Consent Script

Did you receive and have a chance to read the information sheet about the study?

[If no] Ok, we are required to provide this information before the interview/focus group. I will be reading from the document, but please feel free to stop me if you have any questions. [Read information sheet aloud]

[If yes] Ok, I would like to remind you that participating in this interview/focus group is voluntary, there are no penalties for refusing to participate, and you can end the interview/leave the focus group at any time. [Include any other important reminders here.]

Do you have any questions? [Answer questions]

Do you agree to participate in this interview/focus group? [If yes, continue. If no, stop now]

[If applicable] Do you agree for the interview/focus group to be audio/video recorded? [If yes, may record. If no, do not record or end participation if recording is required.]

# Appendix C: Additional Resources on Remote Qualitative Data Collection

Abrams, K. & Gaiser, T. (2017). Online focus groups. In N. FieldingR. Lee & G. Blank The SAGE Handbook of online research methods (pp. 435-449). 55 City Road, London: SAGE Publications Ltd doi: 10.4135/9781473957992.n25

Archibald, M. M., Ambagtsheer, R. C., Casey, M. G., & Lawless, M. (2019). Using Zoom Videoconferencing for Qualitative Data Collection: Perceptions and Experiences of Researchers and Participants. International Journal of Qualitative Methods. <https://doi.org/10.1177/1609406919874596>

Biedermann N. The use of Facebook for virtual asynchronous focus groups in qualitative research. Contemp Nurse. 2018 Feb;54(1):26-34. <https://doi.org/10.1080/10376178.2017.1386072>

Block, E. S., & Erskine, L. (2012). Interviewing by Telephone: Specific Considerations, Opportunities, and Challenges. International Journal of Qualitative Methods, 428–445. <https://doi.org/10.1177/160940691201100409>

Boateng B, Nelson MK, Huett A, Meaux JB, Pye S, Schmid B, Berg A, LaPorte K, Riley L, Green A. Online Focus Groups with Parents And Adolescents with Heart Transplants: Challenges and Opportunities. Pediatr Nurs. 2016 May-Jun;42(3):120-3, 154. [PubMed PMID: 27468513.](https://www.ncbi.nlm.nih.gov/pubmed/27468513)

BURKE, Lisa A.; MILLER, Monica K. Phone Interviewing as a Means of Data Collection: Lessons Learned and Practical Recommendations. **Forum Qualitative Sozialforschung / Forum: Qualitative Social Research**, [S.l.], v. 2, n. 2, May 2001. ISSN 1438-5627. Available at: <http://www.qualitative-research.net/index.php/fqs/article/view/959/2094>.

Drabble, L., Trocki, K. F., Salcedo, B., Walker, P. C., & Korcha, R. A. (2016). Conducting qualitative interviews by telephone: Lessons learned from a study of alcohol use among sexual minority and heterosexual women. *Qualitative social work: QSW : research and practice*, *15*(1), 118–133. <https://doi.org/10.1177/1473325015585613>

Gratton, M.-F., & O’Donnell, S. (2011). Communication technologies for focus groups with remote communities: a case study of research with First Nations in Canada. Qualitative Research, 11(2), 159–175. <https://doi.org/10.1177/1468794110394068>

Irani, E. (2019). The Use of Videoconferencing for Qualitative Interviewing: Opportunities, Challenges, and Considerations. Clinical Nursing Research, 28(1), 3–8. <https://doi.org/10.1177/1054773818803170>

Lo Iacono, Valeria, Symonds, Paul and Brown, David H.K. (2016). Skype as a Tool for Qualitative Research Interviews. Sociological Research Online 21(2)12. <http://www.socresonline.org.uk/21/2/12.html>

Matthews, K. L., Baird, M., & Duchesne, G. (2018). Using Online Meeting Software to Facilitate Geographically Dispersed Focus Groups for Health Workforce Research. Qualitative Health Research, 28(10), 1621–1628. <https://doi.org/10.1177/1049732318782167>

Mealer M, Jones Rn J. Methodological and ethical issues related to qualitative telephone interviews on sensitive topics. Nurse Res. 2014 Mar;21(4):32-7. doi:10.7748/nr2014.03.21.4.32.e1229. PubMed PMID: [24673351.](https://www.ncbi.nlm.nih.gov/pubmed/24673351)

O'Connor, H. & Madge, C. (2017). Online interviewing. In N. FieldingR. Lee & G. Blank *The SAGE Handbook of online research methods* (pp. 416-434). 55 City Road, London: SAGE Publications Ltd doi: 10.4135/9781473957992.n24

Tates, K., Zwaanswijk, M., Otten, R. *et al.* Online focus groups as a tool to collect data in hard-to-include populations: examples from paediatric oncology. *BMC Med Res Methodol* 9, 15 (2009). <https://doi.org/10.1186/1471-2288-9-15>

Wirtz AL, Cooney EE, Chaudhry A, Reisner SL, American Cohort To Study HIV Acquisition Among Transgender Women (LITE) Computer-Mediated Communication to Facilitate Synchronous Online Focus Group Discussions: Feasibility Study for Qualitative HIV Research Among Transgender Women Across the United States. J Med Internet Res 2019;21(3):e12569. URL: <https://www.jmir.org/2019/3/e12569>

Woodyatt, C. R., Finneran, C. A., & Stephenson, R. (2016). In-Person Versus Online Focus Group Discussions: A Comparative Analysis of Data Quality. Qualitative Health Research, 26(6), 741–749. <https://doi.org/10.1177/1049732316631510>
